# O047: The effect of active surveillance culture of carbapenem resistant Acinetobacter baumannii on the occurrence of carbapenem resistant Acinetobacter baumannii bacteremia in a single intensive care unit

**DOI:** 10.1186/2047-2994-2-S1-O47

**Published:** 2013-06-20

**Authors:** G Kim, B Oh, JS Song, PG Choe, WB Park, HB Kim, N-J Kim, EC Kim, M-D Oh

**Affiliations:** 1 Internal Medicine, National Medical Center, Seoul National University Hospital, Seoul, Korea, Republic Of; 2 Infection Control Unit, Seoul National University Hospital, Seoul, Korea, Republic Of; 3 Internal medicine, Seoul National University Hospital, Seoul, Korea, Republic Of; 4 Internal medicine, Seoul National University Bundang Hospital, Seoul, Korea, Republic Of; 5 Laboratory medicine, Seoul National University Hospital, Seoul, Korea, Republic Of

## Introduction

Carbapenem resistant *Acinetobacter baumannii*(CRAB) is an emerging pathogen of healthcare associated infection and little is known about the effectiveness of the active surveillance culture of CRAB.

## Objectives

This study aims at evaluating the effect of active surveillance culture of CRAB upon intensive care unit(ICU) admission on the occurrence of new CRAB bacteremia in ICU.

## Methods

Since February 2011, the active surveillance culture of CRAB was performed in all patients admitted to medical ICU in Seoul national university hospital. Contact precaution was applied for the patients who had positive surveillance culture results. Respiratory specimen were obtained and the isolates were cultured overnight in blood agar plates which contain imipenem. To assess the effectiveness of active surveillance culture of CRAB and contact precaution, the rate of new CRAB bacteremia was compared between baseline period(February 2012-January 2011) and intervention period(June 2011-February 2012). The new CRAB bacteremia case was defined as the CRAB bacteremia occurred later than 48 hours of ICU admission in patients without positive CRAB clinical culture in the prior 12 months and within the 48 hours of ICU admission.

## Results

In the baseline period there were 242 total admissions to the ICU(7355 patients-days) and in the intervention period there were 266 total admissions to the ICU(5432 patients-days). During the intervention period, 21(7.9%) patients showed positive CRAB surveillance culture results. The rate of new CRAB bacteremia is 2.72 cases per 1000 patients-days in the baseline period and 0.92 case per 1000 patients-days in the intervention period(*P*=0.019).

## Conclusion

When active surveillance culture of CRAB and contact precaution for the patients of positive results were applied in a medical ICU, the rate of new CRAB bacteremia was lowered and the time betweennew CRAB bacteremia and ICU admission was lengthened.

## Disclosure of interest

None declared.

